# Establishment in productive occupations and perceived work ability among former students with special educational needs one year after upper secondary education

**DOI:** 10.3233/WOR-220057

**Published:** 2023-05-16

**Authors:** Moa Yngve, Helene Lidström, Helena Hemmingsson, Elin Ekbladh

**Affiliations:** a Department of Health, Medicine and Caring Sciences, Linköping University, Linköping, Sweden; b Department of Special Education, Stockholm University, Stockholm, Sweden

**Keywords:** Job satisfaction, participation, unemployment, young adults

## Abstract

**BACKGROUND:**

Establishment in productive occupations i.e. work and further studies, is challenging for students who experience special educational needs (SEN).

**OBJECTIVE:**

The study aim was to investigate productive occupations and perceived work ability one year after upper secondary education among former students with SEN who had received a student-centred information and communication technology (ICT) intervention.

**METHODS:**

Questionnaire data on productive occupations (*n* = 81) were complemented with the semi-structured Worker Role Interview (WRI) concerning perceived work ability (*n* = 20), in an embedded mixed methods design. Group comparisons between participants who were and were not established in productive occupations were performed. Written notes from the WRI were analysed with a deductive content analysis.

**RESULTS:**

Findings demonstrated that almost two-thirds (63% *n* = 51) of the former students with SEN were established in productive occupations. The established group had to a higher extent obtained pass grades and had to a lesser extent received time-assisting ICT. Managing daily routines in combination with a productive role in a satisfactory manner was perceived as most challenging for the participants in relation to their work ability.

**CONCLUSION:**

The results indicate that students with SEN need person-centred support to handle difficulties both in and outside upper secondary school to promote the transition from school to establishment in productive occupations.

## 1 Introduction

Establishment in the labour market and enrolment in higher education are positive from a health perspective. They increase participation in society and contribute to young people’s well-being [1, 2].

In Europe, the unemployment rate is 12% among young people with graduation from upper secondary education, and 35% for those without graduation [3]. Graduation from upper secondary education is generally considered a prerequisite for successful inclusion in the labour market and entry to higher education [4]. In Sweden, 6% (*n* = 65 000) of young people aged 15–24 were unemployed and not in education or training in 2019. The majority of these individuals were 20–24 years old (*n* = 47 000), representing 8% of the total population in that age group [5], indicating an unsuccessful transition from school to productive occupations related to adult life.

Students with special educational needs (SEN), defined in the Salamanca statement, as students who for various reasons need extra support to achieve educational goals [6], are less likely to graduate from upper secondary education [7–9]. Furthermore, they are overrepresented among unemployed young people who are not in education or training [7, 8, 10]. Low graduation rates, overrepresentation in failed grades and school drop-out among students with SEN contribute to their challenges to becoming established in productive occupations [7, 11, 12], which seems to persist into adulthood [13, 14]. Their school failures have commonly been explained by loss of motivation and high rates of absence from school by leading officials in Swedish municipalities [15]. However, examples of reasons stated by upper secondary school students themselves are commonly related to an unsupportive home and school environment and lack of the necessary adjustments to their educational needs [15–17]. Båtevik [13] concluded that, irrespective of gender, but particularly for women, graduation significantly increases the probability of employment for former students with SEN. Efforts to support students with SEN to obtain educational qualifications should be prioritised to decrease their risk of social and economic vulnerability, health problems [7, 18–20], loss of productive capacity, and costs for the welfare system. In a study by Yngve et al. [21], students with SEN who received a student-centred information and communication technology (ICT) intervention in upper secondary school perceived a significantly more supportive school environment and a positive tendency in terms of increased school attendance.

Past experiences of occupational performance, expectations about the future and the person's ability to handle and organise daily routines have been found to have a significant impact on a person’s work ability and establishment in the labour market [22]. This might imply that the difficulties students with SEN have experienced in their schooling influence their perceived work ability and the productive occupations they enrol in. Persons’ work ability is complex since it relates to both personal and contextual factors both at and outside work [23].

Studies focusing on environmental aspects as either barriers or support to young people’s participation in productive occupations are emphasised [12]. In a recent scoping review about the state of the art in research concerning the transition from childhood to adulthood for people with disabilities, Tideman et al. [12] stressed the need for knowledge about the long-term outcomes of support provided in school. Recently, Myklebust and Båtevik [14] found that support from teacher assistants and special pedagogical support did not contribute to economic independence as adults among former students with SEN. By using a longitudinal approach, which is rare when investigating the transition from school to work among young adults with disabilities [12, 14], this present study strives to provide knowledge about establishment in productive occupations related to adult life among former students with SEN who received assistive technology support in upper secondary school. More specifically, the aim of the study was to describe productive occupations and perceived work ability one year after upper secondary education among former students with SEN who had received a student-centred ICT intervention in upper secondary education.

The following research questions were posed:

– In what types of productive occupations and to what extent do former students with SEN participate?
– Are there differences in demographics, final grades and received ICT support among former students who are established on the labour market or enrolled in higher education and those who are not?
– How do former students with SEN perceive their work ability in relation to their motivation, daily routines and social environment?

## 2 Methods

### 2.1 Design

This descriptive study, which investigates productive occupations and perceived work ability among former students with SEN, was conducted using an embedded mixed methods approach [24]. Quantitative cross-sectional data from a questionnaire was used to investigate productive occupations among the former students, supplemented with qualitative data collected using the Swedish version of the Worker Role Interview (WRI) [25] to describe their perceived work ability. This study has been conducted in accord with the Declaration of Helsinki of 1964 and its later amendments. The Regional Ethics Board in in Linköping, Sweden, gave ethical approval for the study, study codes 2013/409-31 and 2015/203-32.

### 2.2 Participants and data collection

The study population was derived from an earlier study by Yngve et al. [21], evaluating the impact of a student-centred ICT intervention on school participation among 300 upper secondary school students with SEN in Sweden. These 300 students were from all three upper secondary educational levels and attended either a vocational or higher education preparatory programme. Of these 300 students, 244 provided written informed consent and contact information and agreed to be contacted one year after leaving upper secondary school, making them eligible for inclusion in the present study. The data collection was performed during year 2014 until 2018 and consisted of two parts. First, eligible participants were sent the cross-sectional questionnaire, in which they decided whether or not to participate in the WRI interview, which was conducted on a later occasion.

Contact information for 22 students was invalid at the time of data collection, resulting in 222 eligible participants, who were contacted by telephone and/or via email approximately one year after they left upper secondary education. Seventeen students declined participation when approached. The questionnaire was answered by 81 students while 124 did not respond, resulting in a response rate of 36%. No significant differences in demographics (age, diagnosis, educational programme, gender or native language) were found among the 81 participants and the 141 students who did not respond to the questionnaire. Forty-one (51%), of the 81 participants, gave consent to participate in the WRI interview, of whom 20 actually participated. No significant differences in demographics were found among the former students who participated in the WRI interview and the 141 students who did not respond to the questionnaire.

#### 2.2.1 Questionnaire

A form with demographic questions, referred to as questionnaire was created by the research group. The included questions with fixed response options concerned the former students educational programme in upper secondary school, final grades, type of housing, current productive occupation and source of income. Related open-ended questions that required written responses concerned what kind of employment or studies the participant was engaged in and to what extent.

The questionnaire was used during telephone calls or as a web-based version, administrated using survey software. The first author sent a link to the web-based version of the questionnaire as a text message or an email to the telephone number/email address of the potential participant, following up with two reminders where necessary. Of the 81 survey respondents, data was collected via the web-based version for 46 participants and by telephone calls for 35 participants. The first author collected data via telephone, using the questionnaire as a structured interview guide, for 20 participants and two occupational therapists working as research assistants collected data for the remaining 15 participants.

#### 2.2.2 Demographics and ICT intervention

From the above-mentioned study by Yngve et al. [21], demographics (age, diagnosis, gender and native language) and information concerning the ICT intervention (device and software) that students received during their upper secondary education were retrieved. The ICT devices were a computer/laptop, a tablet and/or a smartphone and different types of software, categorised as ‘time-assisting’ (calendar, schedule, reminder, timer) or as supporting ‘writing and reading’ (word processor, text reader, scanner, recorder).

#### 2.2.3 The Worker Role Interview (WRI)

The WRI-S [25] is a semi-structured interview and therapist-administered assessment, conceptually based on the Model of Human Occupation (MOHO) [26]. The WRI is used to identify how a person’s motivation, daily routines and the surrounding social environment affect the person’s work ability by investigating 16 items related to six theoretical MOHO concepts: Personal causation, Values, Interests, Roles, Habits and Environment. The interviewer takes notes during the interview and rates the 16 items on a four-point rating scale after the interview. The scores 1 (strongly interferes) and 2 (interferes) imply that the content related to the actual item interferes with the individual’s work ability and a score of 3 (supports) or 4 (strongly supports) implies that the item constitutes a support for the individual’s work ability. If an item is not applicable or not enough information has been collected to perform the rating of an item, the rating ‘NA’ (not applicable) is used. As well as the rating itself, notes explaining the unique personal reasons for the chosen rating are written on the rating form for each item. Several psychometric studies have concluded that the Swedish version of the WRI is psychometrically sound [22, 27–29].

Participants who gave consent on the questionnaire to participate in the WRI interview were contacted by the first author or research assistants via telephone to schedule a time. At least two reminders were used. The semi-structured interview was adapted based on each respondent’s answers and situation. The first author performed 15 WRI interviews and the research assistants performed the other five. During the interviews, notes and statements to recall what the participant said in relation to the content of the WRI were documented. The interviewer completed the rating form and wrote explanatory notes for every item in connection with each WRI interview. The explanatory notes on the rating form could consist of both statements and a synthesis of what the participant had said in relation to the specific item. The rating ‘NA’ was used when, for example, items were related to a specific work environment (items 13, 15–16) and the participant was without a job. The interviews conducted by the first author lasted between 22 and 50 minutes (*M* = 33 minutes). No time range for the five WRI interviews performed by the research assistants was available.

### 2.3 Data analysis

Quantitative, cross-sectional data consisting of responses to the questions from the questionnaire and ratings of WRI items was analysed using descriptive statistics [30]. In the present study, productive occupations are defined as including paid and unpaid activities [26] which in turn include paid employment, studying, and activities to improve opportunities for employment, such as labour market training and work experience placement. Participants were divided into two groups based on the type and extent of their reported productive occupation. Participants who reported that they on a weekly basis worked 50% or more of full time, were enrolled in higher education or who currently were on parental leave from a specific workplace (*n* = 1) were considered to be ‘Established in work or higher education’. ‘Not established in work or higher education’ included participants who were working less than part time (50%) on a weekly basis, were unemployed/seeking employment, enrolled in adult education equivalent to upper secondary education, or were receiving compensation from the Swedish social insurance system. Fisher’s exact tests [30] was conducted to investigate the differences between the two groups in terms of demographics, final grades and type of ‘time-assisting’ or ‘supporting writing and reading’ ICT devices received during the intervention in upper secondary education. Analyses were performed in SPSS, version 24 [31], and the significance level was set at *p* < .05.

The written notes on the WRI rating forms constituted the unit of analysis in the qualitative content analysis. Since the WRI is theoretically based on concepts in the MOHO, a deductive approach was followed, guided by Graneheim, Lindgren and Lundman [32]. A deductive analysis moves from theory to the concrete data level [32] and concepts from the MOHO were used to guide the analysis of which aspects influenced participants’ perceived work ability. The six MOHO concepts in the WRI were used as content areas and were, together with their theoretical definitions, inserted into matrices of the related theoretical constructs of motivation, daily routines and social environment, which were used as categories. For each content area, the manifest content of the written notes related to the included WRI items was assembled. As an example, the manifest content from the items ‘Assesses abilities and limitations’, ‘Expectations of job success’ and ‘Takes responsibility’ was assembled in the content area ‘Personal causation’ located under the category ‘motivation’. The content of each area was read through several times by the first author to obtain a sense of the whole. The process of abstraction began with identifying meaning units in the written notes. In most cases, the written notes were abstracted to even shorter, concrete meaning units, which were then coded. Meaning units that concerned the same thing, irrespective of whether they were considered as supportive or hindering factors for the person's work ability, received the same code, inspired by the theoretical concepts. The analysis and process of abstraction were performed with a word processor program by using matrices and highlighting the text in different colours. In the process of abstracting codes in relation to the concepts, the first and last author engaged in dialogue to reach consensus.

## 3 Results

Demographics and the questionnaire data of the 81 former students with SEN are presented in Table 1, divided into the two groups: ‘Established in work or higher education’ and ‘Not established in work or higher education’. Two-thirds (*n* = 55) of the participants had studied a vocational programme and 51% (*n* = 41) were male. Fifty-six percent (*n* = 45) of the participants had a medical diagnosis and 51% (*n* = 41) reported that they had achieved passing grades in all subjects included in their upper secondary programme. Sixty-one percent (*n* = 49) lived with their guardians.

**Table 1:** Demographics and information from the questionnaire concerning participants’ productive occupations (n = 81)

	Whole group, *n* = 81	Established in work or higher education, *n* = 51, 63%	Not established in work or higher education, *n* = 30, 37%	Fisher’s exact test, *p*
Gender, *n* = 81^A^				.36
Male	41 (51)	28 (55)	13 (43)
Female	40 (49)	23 (45)	17 (57)
Diagnosis, *n* = 81^A^				.11*
No diagnosis	36 (44)	19 (37)	17 (57)
Diagnosis	45 (56)	32 (63)	13 (43)
Neuropsychiatric disorder	13 (16)	8 (16)	5 (16)
Dyslexia	23 (28)	17 (33)	6 (20)
Other	9 (11)	7 (14)	2 (7)
ICT, *n* = 81^A^
Time-assisting	59 (73)	32 (63)	27 (90)	.01
Writing and reading	58 (72)	36 (71)	22 (73)	.79
Educational programme, *n* = 81				.86
Vocational	55 (68)	35 (69)	20 (67)
Preparatory	26 (32)	16 (31)	10 (33)
Final grades, *n* = 81				.02
Pass in all subjects	41 (51)	29 (57)	12 (40)
Pass in most subjects	31 (38)	20 (39)	11 (37)
Pass in some or no subjects	9 (11)	2 (4)	7 (23)
Productive occupation, *n* = 81
Work	45 (56)	43 (84)	2 (7)^B^
Job-seeking	12 (15)	7 (14)	12 (40)
Adult education^C^	10 (12)	1 (2)	10 (33)
Higher education	7 (9)		6 (13)
Other^D^	7 (9)
Occupied on a weekly basis, *n* = 58	n=46	n=12	.32
Full-time	37 (64)	31 (67)	6 (50)
Less than full-time	21 (36)	15 (33)	6 (50)
Type of housing, *n* = 81				.48**
Parent/guardian	49 (61)	29 (57)	20 (67)
Independent	30 (27)	22 (43)	8 (27)
Other	2 (2)		2 (7)

Note: ^A^Data retrieved from study by Yngve et al. (2021). ^B^Working less than half time. ^C^Equivalent to upper secondary education. ^D^Parental (*n* = 1) or sick leave (*n* = 2), work experience placement (*n* = 2), or travel (*n* = 2). ^*^Dichotomised as Diagnosis or No diagnosis. ^**^Dichotomised as Independent living or Other.

### 3.1 Productive occupations

Most participants had become ‘Established in work or higher education’ (*n* = 51, 63%), with 43 participants working, seven enrolled in higher education and one being on parental leave from a specific workplace (see Table 1). In the group ‘Not established in work or higher education’ (*n* = 30, 37%), the largest proportion of participants were seeking employment (*n* = 12, 15%) or enrolled in adult education equivalent to upper secondary education (*n* = 10, 12%). On a weekly basis, 64% of the participants (*n* = 37) reported full-time engagement in their current occupation, see Table 1.

### 3.2 Differences between participants ‘established in work or enrolled in higher education’ and ‘not established in work or enrolled in higher education’

Participants who were established in work or enrolled in higher education reported final grades in all subjects to a higher extent (*p* = .02 *Fisher*’*s exact test*), and had less often received time-assisting ICT (*p* = .01 *Fisher*’*s exact test*) than those who were not established in work or higher education. No differences between the groups were found in relation to demographics, or received ICT for writing and reading, see Table 1.

### 3.3 Participants’ perceived work ability

Eleven of the 20 participants who participated in the WRI interview were boys and 13 of them had become ‘Established in work or higher education’. The ratings of the participants’ WRI items are presented in Table 2. The WRI item ‘Commitment to work’ was the only item rated as supportive among all participants, indicating that work was a valued occupation for all. The item ‘Pursues interests’ was the item rated as most unsupportive in relation to participants’ psychosocial work ability, as almost more than a third of the participants had that item rated as interfering, which indicates that these participants experienced difficulties in finding ways to be satisfied with their daily lives, see Table 2. Items related to participants’ ‘Roles’ in and outside their productive occupation were rated as supportive for work ability for all but one participant. In regard to habits, also related to the content area ‘Daily routines’, hindering aspects in ‘Daily routines’ that interfered with perceived work ability were found by four of the participants. Items within the content area ‘environment’ had drop-out in several items because some participants did not have a specific workplace to relate to, but the item was for the most part found to be supportive of participants’ work ability. The WRI item that interfered most was related to participants’ perceptions of their manager, which was rated as interfering by three participants.

**Table 2:** Ratings of WRI items by the 20 participants, sorted under the content areas and categories derived from the MOHO, with the associated codes identified in the deductive content analysis

Categories	WRI content areas	WRI items	Interferes		Supports			Codes
			1	2	3	4	NA*
Motivation
	Personal causation	Assesses abilities and limitations	1	2	1	16		•*Compliance between capacities and demands* •*Goals for the future* •*Efforts to achieve desired ends*
		Expectation of job success	1	1		15	3
		Takes responsibility	2	1	3	13	1
	Values	Commitment to work			9	11		•*Valuing productive occupations*
		Work-related goals	1	3	3	13
	Interests	Enjoys work	1		4	11	4	•*Stimulated by productive occupation* •*Desire for change*
		Pursues interests	2	5	1	10	2
Daily routines	Roles	Appraises work expectations		1	3	14	2	•*Internalised productive role* •*Balancing of roles*
		Influence of other roles		1		15	4
	Habits	Work habits		2	4	10	4	•*Maintain daily routines* •*Disability impacted routines*
		Daily routines	3	1	2	12	2
		Adapts routine to minimise difficulties	1	2		12	5
Social environment	Environment	Perception of work setting	1	2	3	6	8	•*Social support at work* •*Social support from family/friends*
		Perception of family and peers		2	1	15	2
		Perception of boss	1	2	1	7	9
		Perception of co-workers		1	2	10	7

Note: *NA=Not applicable or not enough information to rate.

The six theoretical constructs included in the WRI that constituted the content areas were sorted under the three categories of motivation, daily routines and social environment, derived from the MOHO. The associated codes for each content area are listed in Table 2 and written in italics in the text under the following headings.

#### 3.3.1 Motivation for productive occupations

Motivational aspects with influence on participants’ perceived work ability were sorted into six codes. Most participants stated that they felt in control and did not experience any limitations in relation to their current occupation and described how they experienced a *compliance between their capacities and the demands* of their productive occupation. They perceived themselves to have the ability, with or without support, to handle their productive occupation and to perform with good results. Many who had experienced limitations previously said that they had developed strategies to manage their occupation; for example, they used ICT (tablets and smartphones) to support the structuring and planning of daily activities, or to conduct study-related tasks. Aspects described as lowering motivation were related to participants’ self-doubt about their ability to manage a ‘regular job’ and being controlled by their disability or outside factors. One participant described this by saying the disability sometimes took the upper hand over strategies that normally made studying manageable. All participants’ expressed *goals for the future*, in terms of plans related to productive occupations and overall were optimistic about their future. However, their *efforts to achieve desired ends* varied. Examples considered to be supportive of participants’ work ability involved complementing grades in specific courses in adult education (equivalent to upper secondary education) to be able to study at the university, and wanting full-time employment or employment in the field of their education that motivated them in job-seeking, even though some already had a job. Lack to initiate change in their occupational situation, even though participants wanted something else as their future occupation, was considered to interfere with their work ability. The participants were *valuing productive occupations* as it generated work experience or qualifications for further studies, or to earn money. Furthermore, a productive occupation contributed to structuring the day, their well-being and feelings of being needed. Most of the participants were *stimulated by their productive occupation*; they expressed feelings of enjoyment in relation to work or study and found their occupation self-developing, or enjoyed interactions with colleagues, customers and study peers. Most participants identified interests they liked to engage in during their leisure time, such as spending time with friends and family or engaging in physical exercise. However, participants also expressed a *desire for change.* Some participants stated that they did not have enough time to spend on hobbies or felt that their working hours or their disability prevented them from engaging in their interest to a satisfying extent. A few stated that they did not have any interests or expressed feelings of loneliness.

#### 3.3.2 Daily routines in relation to work and further studies

This category constitutes four codes with influence on participants’ perceived work ability. Most participants had *internalised their productive role*; they were well aware of how to behave in their occupational role and gave examples of expected behaviour, such as being ‘service-minded’. They knew how to execute tasks and what to consider during interactions with people they encountered in their role. Overall, the participants’ statements implied that they experienced a *balance between roles.* Many lived with their guardians and still held their old role as an adolescent, which enabled them to prioritise and combine their productive role with other roles in a satisfactory manner. In contrast, participants in part-time employment stated that their working role could have a negative impact on other roles. They accepted additional working hours when offered, which complicated balancing their working role with their other roles. As regards *maintain daily routines*, the participants’ routines in and outside work or studies were in general described as satisfactory and supportive of their work ability. Some expressed the importance of structure in order to combine their productive occupation and daily routines outside work, and used support such as calendars and reminders to plan the week and their commitments. However, maintaining an occupational role required much effort for some participants who said that their productive role consumed too much time; their everyday habits were disrupted and it was difficult to plan and maintain their everyday life and daily routines outside work or study. Furthermore, some participants experienced that *disability negatively impacted routines* and felt it hindered both their working and daily routines. When they felt stressed or ill, their routines were ineffective, which in turn negatively affected their well-being.

#### 3.3.3 Participants’ perception of the social environment influence on work ability

Two codes were identified in relation to social environmental aspects with an influence on participants’ perceived work ability. The majority of the participants who were working experienced *social support at work* and did not perceive any barriers to their occupational performance. Most stated that they had confidence in their employer/manager, who had listened to them when they needed support or when improvements needed to be made in their work environment. However, there were also those who lacked confidence in their manager’s ability to give feedback or enable fair opportunities for part-time employed staff, which was considered as an interfering aspect in relation to their perceived work ability. For example, one manager did not allow part-time employed staff to attend on-the-job education/training, which made the participant feel overlooked. Participants stated that they knew they could rely on *social support from family and/or friends* when they needed it, both in the process of obtaining employment or enrolling in studies and in maintaining these roles. For example, there were participants who said that family members had arranged contacts with employers who were looking to hire new staff; others said that family members had offered emotional support when ‘things were tough’. Hindering aspects involved statements about wanting more support from family and/or friends than participants had received, and the feeling of being alone in the process of finding a job.

## 4 Discussion

The aim of the present study was to describe productive occupations and perceived work ability one year after upper secondary education among former students with SEN who had received an ICT intervention in upper secondary education. A rather high proportion of the participants (63%, *n* = 51) had become established in productive occupations one year after upper secondary school, compared to other studies reporting an employment rate of about 50% among former students with SEN in their twenties [9, 33]. Considering that vocational experience and early entry into the labour market are associated with future employment among former students with SEN [7, 9], the present finding is considered positive. However, few (*n* = 7) of the present participants were enrolled in higher education. One explanation could be that the majority (68%) of participants had studied within a vocational programme, which among students with SEN has been associated with a low likelihood of enrolling in further studies after upper secondary education [8].

The group of participants who were established in productive occupations had to a higher extent obtained final grade in all subjects, confirming that graduation from upper secondary education is a predictor of both employment and enrolment in higher education [11]. Furthermore, the established group had received time-assisting ICT to a lesser extent than the group of participants who were not established in work or higher education. These findings might suggest that the established group of students were academically successful and not hindered by difficulties related to time management and planning or conducting tasks, indicating that they might have fewer functional challenges in comparison to the other group of former students, and thus better opportunities in relation to enrolment in work or further studies. These results are in line with the findings of Båtevik [13] and Myklebust and Båtevik [14], who concluded that the degree of functional challenge is related to long-term employability among students with SEN. On the other hand, this finding might also indicate that students with difficulties related to time management, which is common among students with neuropsychiatric disorders and/or behavioural challenges [34] are a more vulnerable group. Not only do they struggle academically to a great extent, but they also encounter greater challenges in the transition to productive occupations [35]. The need for both academic and psychosocial support during schooling has been stressed by students with neuropsychiatric disorders [36] and their need for individualised support and services in the transition to productive occupations after upper secondary school has been emphasised [37, 38]. As such, how to best support students with difficulties in time management in relation to activities, both at school and in the process of establishment in work and further studies, is still an area that warrants further investigation. The characteristics of the group of former students with SEN who were not established in productive occupations may have implications for professionals supporting young adults in their transition to productive occupations related to adult life.

The qualitative findings showed that participants were optimistic about their future and believed in their work ability, although they had experienced support needs in upper secondary school. Among former students with SEN, self-advocacy has been identified as a factor associated with employment [39]. Several of the participants gave examples of strategies or the use of ICT to manage their productive occupation and/or daily routines, suggesting that they possessed knowledge about how to accommodate their difficulties. In the study by Lundahl et al. [16], the former students with SEN enrolled in adult education still perceived a lack of support and were not able to complete the courses to obtain a complete upper secondary education.

Hindering aspects mentioned by the participants were primarily related to their daily routines outside a productive role, as they struggled with managing daily routines in combination with a productive role in a satisfactory manner. The findings contribute to the understanding of how motivation, daily routines and the surrounding social and physical environment influence work ability among former students with SEN. The results indicate that students with SEN need person-centred support to handle difficulties that may arise in the interaction between the person and the environment, both within and outside upper secondary school, to promote the transition from school to establishment in productive occupations.

### 4.1 Methodological considerations

Some methodological considerations need to be addressed concerning the drop-out rate and trustworthiness of the qualitative findings. A longitudinal approach is often associated with an increased risk of a high drop-out rate and this study had a drop-out rate over 60%. Part of the explanation for this might be potential participants’ unwillingness to answer phone calls from unknown telephone numbers, or that their contact information had been collected several years previously. Measures were taken to combat this issue by using both phone calls and a web-based version of the questionnaire, with reminders. However, if participants had changed their telephone number or email address, these measures were inadequate. Access to potential participants’ personal identity numbers would have provided better opportunities to make contact with individuals who had consented to participate in the present study.

Furthermore, the small number of former students who participated in the WRI interview made it impossible to statistically compare if there were differences in the WRI ratings between those who were established or not in productive occupations. The credibility of the qualitative findings might have been strengthened because of the heterogeneity in the data, including WRI interviews with participants of both genders with experiences of SEN during upper secondary education, and who were and were not established in work or higher education. Credibility is also about the data collected and the appropriateness of the collection method [40]. Using the WRI, which was designed to investigate perceived work ability, provided a structured method to collect relevant information to investigate participants’ perceived work ability in terms of motivation, daily routines and social environment. Furthermore, using the MOHO to guide the content analysis provided a valid frame of reference and was considered helpful since it offered a theoretical explanation for how motivation, daily routines and the surrounding environment influence an individual’s perceived work ability [26]. However, an already processed text, such as the notes in the WRI rating forms, limits the possibility of abstraction and interpretation [41] and the analysis and presentation of the results was therefore kept at a descriptive level, which remained close to the participants’ statements. As pointed out by Hsieh and Shannon [42], a deductive approach might lead to new insights being missed. Therefore the first and last authors engaged in an ongoing dialogue during the process of analysis and formulation of findings, to ensure correspondence between the data and the theoretical model. Another concern, related to dependability [32], is the fact that there were three different WRI interviewers. However, both the first author and the research assistants had knowledge of the MOHO and were trained users of the WRI with its semi-structured nature, which might have strengthened the dependability of the collected data.

## 5 Conclusion

Almost two-thirds of the former students with SEN were established in productive occupations one year after upper secondary education. Participants who had obtained final grades in all subjects and had not received time-assisting ICT in the intervention in upper secondary school were established in work or further education to a higher extent. Some participants struggled with maintaining their daily routines and the balance between their different roles to obtain a satisfactory everyday life situation. Thus, the difficulties students experience concerning managing time in relation to performance of, and participation in activities needs to be carefully addressed. It is important that these aspects are considered by student health units at upper secondary school as they seem to have a decisive role in the transition from school to establishment in productive occupations for students with SEN.

## Ethical approval

The study was approved by the Regional Ethics Board in Linköping, Sweden (study codes 2013/409-31 and 2015/203-32).

## Informed consent

Written informed consent was collected from all participants.

## Conflict of interest

The authors declare that they have no conflicts of interest.
